# Electrophoretic Deposition and Characterization of Thin-Film Membranes Li_7_La_3_Zr_2_O_12_

**DOI:** 10.3390/membranes13050468

**Published:** 2023-04-27

**Authors:** Efim Lyalin, Evgeniya Il’ina, Elena Kalinina, Boris Antonov, Alexander Pankratov, Danil Pereverzev

**Affiliations:** 1 Laboratory of Electrochemical Power Sources, Institute of High Temperature Electrochemistry, Ural Branch of the Russian Academy of Sciences, 620990 Yekaterinburg, Russia; efim.lyalin.2013@inbox.ru (E.L.); ilyina@ihte.uran.ru (E.I.); antonovbd@ihte.uran.ru (B.A.); a.pankratov@ihte.uran.ru (A.P.); 2 Laboratory of Complex Electrophysic Investigations, Institute of Electrophysics, Ural Branch of the Russian Academy of Sciences, 620016 Yekaterinburg, Russia; 3 Department of Physical and Inorganic Chemistry, Institute of Natural Sciences and Mathematics, Ural Federal University, 620002 Yekaterinburg, Russia; 4 Laboratory of Solid State Ionics, Institute of Solid State Chemistry, Ural Branch of the Russian Academy of Sciences, 620108 Yekaterinburg, Russia; danil_per@mail.ru

**Keywords:** all-solid-state batteries, electrophoretic deposition, thin-film electrolyte membrane, Li_7_La_3_Zr_2_O_12_, lithium-ion conductivity

## Abstract

In the presented study, films from tetragonal Li_7_La_3_Zr_2_O_12_ were obtained by electrophoretic deposition (EPD) for the first time. To obtain a continuous and homogeneous coating on Ni and Ti substrates, iodine was added to the Li_7_La_3_Zr_2_O_12_ suspension. The EPD regime was developed to carry out the stable process of deposition. The influence of annealing temperature on phase composition, microstructure, and conductivity of membranes obtained was studied. It was established that the phase transition from tetragonal to low-temperature cubic modification of solid electrolyte was observed after its heat treatment at 400 °C. This phase transition was also confirmed by high-temperature X-ray diffraction analysis of Li_7_La_3_Zr_2_O_12_ powder. Increasing the annealing temperature leads to the formation of additional phases in the form of fibers and their growth from 32 (dried film) to 104 μm (annealed at 500 °C). The formation of this phase occurred due to the chemical reaction of Li_7_La_3_Zr_2_O_12_ films obtained by electrophoretic deposition with air components during heat treatment. The total conductivity of Li_7_La_3_Zr_2_O_12_ films obtained has values of ~10^−10^ and ~10^−7^ S cm^−1^ at 100 and 200 °C, respectively. The method of EPD can be used to obtain solid electrolyte membranes based on Li_7_La_3_Zr_2_O_12_ for all-solid-state batteries.

## 1. Introduction

Solid electrolytes with lithium-ion conductivity are widely used in high-temperature electrochemical energy devices. Moreover, they are considered promising membranes for lithium low- and intermediate-temperature all-solid-state batteries (ASSB) [1,2]. Currently, the development of methods for obtaining thin-film structures of various functional materials suitable for practical application is one of the most important tasks of modern science and technology. The films of solid electrolytes and the electrochemical devices based on them are in great demand since the transition to film structures will make it possible to miniaturize power sources and save often expensive materials in mass production. Moreover, the thickness of the electrolyte membrane has a significant influence on the electrochemical characteristics of ASSB [3]. Wet-chemical and vacuum-based methods are most often used to obtain the films of lithium-conducting solid electrolytes [4,5].

Li_7_La_3_Zr_2_O_12_ (LLZ) and compounds based on it are considered one of the most promising solid electrolytes for use in ASSBs [6,7,8,9]. LLZ has two modifications: cubic with a lithium-ion conductivity of ~10^−^^4^ S cm^−^^1^ at room temperature and tetragonal, characterized by lower values of total conductivity (~10^−^^6^–10^−^^7^ S cm^−^^1^) [9]. It is well known that doping of LLZ on various sublattices, with the cations of different elements, for example, Al^3+^, Ga^3+^, Ta^5+^, etc., is used to stabilize the cubic phase. In the literature, various methods for obtaining thin films of LLZ solid electrolytes on different substrates (steel, Pt, Ti, Si, MgO, Al_2_O_3_, etc.) are presented [5,9,10,11,12,13,14,15]. However, such problems as microcracks, impurity phases due to the loss of lithium during annealing of ultrathin electrolyte films, and underestimated values of lithium-ion conductivity occur during LLZ film formation.

Vacuum methods for obtaining thin films of lithium-conducting solid electrolytes with garnet structures are most frequently mentioned in the literature. The composition of the target must be carefully chosen in order to obtain the film with the composition desired. An excess of lithium-containing components is often used. In these methods, the main phase is formed during annealing; X-ray amorphous LLZ and lanthanum zirconate are formed first. In work [10], the thin films of Li_5_La_3_Ta_2_O_12_ were obtained by a sputter deposition process on different substrates (Y_3_Al_5_O_12_, MgO, SiO_2_, steel). The total conductivity of the film obtained was equal to 1.2·10^−9^ S cm^−^^1^ for a sample deposited on Au-coated steel at 500 °C. LLZ films doped with Al and Ta were obtained by sputtering, for example, by pulsed laser deposition followed by annealing at temperatures up to 1000 °C on SrTiO_3_ and sapphire substrates [11]. The films deposited at room temperature had an amorphous structure and exhibited a lithium-ion conductivity of 3.35·10^−7^ S cm^−^^1^. The films annealed have a higher value of lithium-ion conductivity, 7.36·10^−7^ S cm^−^^1^ [11]. Thin films of Ta, Al-doped LLZ on Si_3_N_4_/Si substrate obtained by the same method in work [5] also have an amorphous structure and low values of conductivity at room temperature (2.86·10^−9^–1.11·10^−13^ S cm^−^^1^). Films of similar composition obtained by magnetron sputtering on stainless steel substrates had low conductivity values too, 2.0·10^−^^9^ S cm^−^^1^ [12]. In work [13], the aerosol deposition method was used to obtain films of cubic garnet solid electrolyte Al_y_Li_7−3y-_zLa_3_Zr_2-z_Ta_z_O_12_ with a thickness of several μm on a silicon substrate. The conductivity of the as-deposited film at room temperature was equal to 2·10^−7^ S cm^−^^1^, but its sintering at 600 °C leads to a significant increase in the conductivity of the solid electrolyte membrane, 7·10^−5^ S cm^−^^1^.

Wet-chemical methods are also actively used to obtain LLZ films [9]. R.J. Chen et al. [14] successfully fabricated LLZ films by sol–gel spin coating on Si substrates with layers of SiO_2_, Ti, and Pt. The films annealed at 600 °C had the highest conductivity values of 1.67·10^−6^ S cm^−^^1^. Moreover, thin films of Al-doped LLZ were obtained on MgO substrates by a dip-coating process, and after annealing, their ionic conductivity was equal to 2.4·10^−6^ S cm^−^^1^ at 25 °C [15].

Electrophoretic deposition (EPD) is a promising method for the formation of thin-film coatings of complex composition with a thickness from ~1 μm to tens of μm on porous and dense substrates of various shapes [16,17]. The EPD method is based on the phenomenon of electrophoresis in a suspension of particles. The movement of particles in the suspension is directed towards the electrode, which leads to their deposition on the electrode and coating formation [18,19]. The coating obtained is dried. Then it can be annealed or sintered. The EPD method is used in the creation of high-temperature electrochemical devices, such as solid oxide fuel cells (SOFCs), namely, in the formation of solid electrolyte layers, for example, based on doped zirconium dioxide and doped cerium dioxide, as well as barium cerate [20,21,22]. The substrates used should possess electrical conductivity for the implementation of EPD; however, it is possible to deposit coatings on non-conductive substrates by creating a conductive sublayer on their surface, for example, graphite or conductive polymer–polypyrrole [23,24,25]. For non-conductive substrates with a sufficient level of through porosity, it is possible to carry out direct EPD due to the appearance of conductive channels in the substrate’s porous structure. In this case, the coating is formed on the substrate’s front surface, which is placed on the reverse side of the electrode immersed in the suspension [26,27]. The distinctive feature of the EPD method is the relationship between the properties of the coating and the dispersed composition of powder in suspension, which can be controlled by such methods as ultrasonic treatment (UST) and centrifugation.

The application of the electrophoretic deposition method for the formation of electrodes for Li-ion batteries is mainly presented in the literature [28]. In the present study, the formation of the thin-film Li_7_La_3_Zr_2_O_12_ membrane on a Ti substrate by electrophoretic deposition has been shown for the first time. The Ti substrate was chosen due to the possibility of its heat treatment at sufficiently high temperatures. The study includes the investigation of electrokinetic zeta potential in a non-aqueous suspension of microsized LLZ powder, the influence of molecular iodine addition; the effect of EPD modes on the thickness of coatings, and the influence of heat treatment conditions on the phase composition, microstructure, and conductivity of the ceramic membrane.

## 2. Materials and Methods

### 2.1. Synthesis and Characterization of the Electrolyte

Li_7_La_3_Zr_2_O_12_ was synthesized by the citrate-nitrate method. Li_2_CO_3_ (99.4%, Reakhim, Moscow, Russia), La_2_O_3_ (99.9%, Vekton, St. Petersburg, Russia), and ZrO(NO_3_)_2_·2H_2_O (98.9%, KhimReaktivSnab, Ufa, Russia) were used as initial compounds. The components were mixed in the stoichiometric ratio, except lithium carbonate, which was taken with an excess of 10 wt% [9]. These reagents were dissolved in a mixture of diluted nitric and citric acids. The resulting mixture was evaporated at 80 °C, and the obtained gel was dried and heated at ~200 °C. The synthesis of powder was performed by increasing the temperature stepwise (700 °C—1 h; 800 °C—1 h; 900 °C—1 h) in an air atmosphere using a high-temperature furnace. The synthesis is described in detail in our previous work [29].

The powder was examined by X-ray diffraction (XRD) with a Rigaku D-MAX-2200V diffractometer (Rigaku, Tokyo, Japan) at room temperature. A curved graphite crystal was used to monochromate CuKα radiation. The data were collected over a 2ө range of 10–60° in continuous mode at a scan rate of 3° min^−1^. High-temperature XRD analysis of the LLZ powder was carried out in the temperature range of 50−700 °C using the Rigaku D-MAX-2200V diffractometer.

The ceramic grain size of the synthesized powder was estimated by scanning electron microscopy (SEM) and Brunauer–Emmett–Teller (BET) analysis. The specific surface area of the powder was determined on the META SORBI N 4.1 (Meta, Novosibirsk, Russia). The SEM study of the powder obtained was carried out using a MIRA3 FEG SEM (Tescan, Brno-Kohoutovice, Czech Republic).

### 2.2. Preparation and Characterization of LLZ Suspensions for EPD

A suspension based on LLZ powder with a concentration of 10 g/L was prepared in a mixed dispersion medium of isopropanol/acetylacetone in a ratio of 70/30 vol.%. The composition and ratio of the dispersion medium were chosen based on our previous investigations [25,30]. UST of LLZ suspension was carried out for 5–125 min at 25 °C using an ultrasonic bath (UZV-13/150-TH). Molecular iodine (0.4 g/L) was added to the suspension to obtain a continuous LLZ coating. Electrokinetic zeta potential and pH in LLZ suspensions were measured by electroacoustic methods using a DT-300 analyzer (Dispersion Technology, Lakewood, NJ, USA).

### 2.3. Electrophoretic Deposition and Characterization of LLZ Films

Electrophoretic deposition of LLZ coatings was carried out using specialized automated equipment (IEP UB RAS, Russia) in a constant voltage mode. Current measurements during deposition were made with a UNI-T UT71E digital multimeter (Uni-Trend Technology, Dongguan, China). EPD of LLZ coating was carried out on an electrode substrate (Ni-foil or Ti-foil, 10 × 20 mm). A stainless-steel plate with the same dimensions was used as the counter electrode. The distance between the electrodes was equal to 10 mm. The EPD process was carried out in a 40 mL suspension without stirring in a beaker with electrodes immersed in the suspension.

The surface morphology of the LLZ coatings deposited was studied using an optical microscope, ST-VS-520 (STAT, Ekaterinburg, Russia). The thickness of dried coatings after EPD was estimated according to their weight and theoretical density of LLZ.

LLZ coatings on Ti substrates were annealed in air at temperatures of 300, 400, and 500 °C and in Ar at 500 °C with an isothermal exposure of 0.5 h using a high-temperature furnace. The samples obtained were examined by the XRD method at room temperature.

An SEM study of the sample surface was carried out using a MIRA3 FEG SEM (Tescan, Brno-Kohoutovice, Czech Republic) in backscattered electron (BSE) and secondary electron (SE) modes. The element distribution study was performed using SEM-EDS (JEOL JSM 5900LV Scanning Electron Microscope with Oxford Instruments INCA Energy Dispersive Spectrometer, Richland, WA, USA).

The phase composition of the film annealed at 500 °C was studied using a Raman spectrometer U 1000 (Renishaw, New Mills, UK). The Raman spectra were collected by an Ar-ion laser with a wavelength of 532 nm and a power of 40 mW at a scanning angle of 90°. The Raman spectra of the sample surface were recorded in the static and extended modes in the range of 1200−50 cm^−1^ with a spectral resolution of 1 cm^−1^. The intensities were normalized to the maximum value.

The thermal properties of the film annealed at 500 °C were studied using simultaneous thermal analysis (STA). The STA measurements were performed in the Pt pans with a heating rate of 10 °C min^−1^ in air and an expulsion rate of 20 mL min^−1^ in the temperature range of 35−900 °C using a thermal analyzer STA 449C Jupiter (Netzsch, Selb, Germany). The results obtained were processed by the NETZSCH Proteus software.

### 2.4. Characterization of Electrochemical Properties of Annealed LLZ Coatings—Impedance Spectroscopy

The resistances of the films obtained (initial and annealed at 300, 400, and 500 °C) were determined by electrochemical impedance spectroscopy. Pt electrodes (S = 0.095 cm^2^) were deposited on the film surface using a Q150T S/E/ES sample preparation system for electron microscopy (Quorum Technologies, Headquarters, UK) by magnetron sputtering from a pure metal target. The measurements were carried out using an E7-25 immittance meter (MNIPI, Minsk, Belarus) in the frequency range of 0.025–1000 kHz in the temperature range of 25–300 °C in an electrochemical cell with silver current leads in air. The total conductivity of ceramics was calculated taking into account the geometric parameters of the measured samples—film thickness and electrode area.

To determine the thickness of film electrolytes annealed at different temperatures, their cross-sections were prepared after the resistance measurements. The samples were placed in a container and filled with epoxy resin with a hardener (NPK Astat—EDP, Dzerzhinsk, Russia) in a ratio of 2.5/1, respectively. After hardening, the recording surface was leveled using a grinding wheel with a diamond grit of 200/160 µm. The final polishing of the samples was carried out using sandpaper with grits (P) 3000 and 5000. To improve the conductivity of the samples, carbon was sprayed onto the prepared surface using a Quorum Technologies Q150R ES sputtering unit. The cross-section of the samples was studied using a scanning electron microscope VEGA 3 LMH (TESCAN, Brno-Kohoutovice, Czech Republic) with 100× and 500× magnifications in SE (10 keV; 30 pA) and BSE (20 keV; 300 pA) modes.

## 3. Results and Discussions

### 3.1. Characterization of the Initial Powder of Electrolyte and Suspensions Based on It

**Figure 1:**
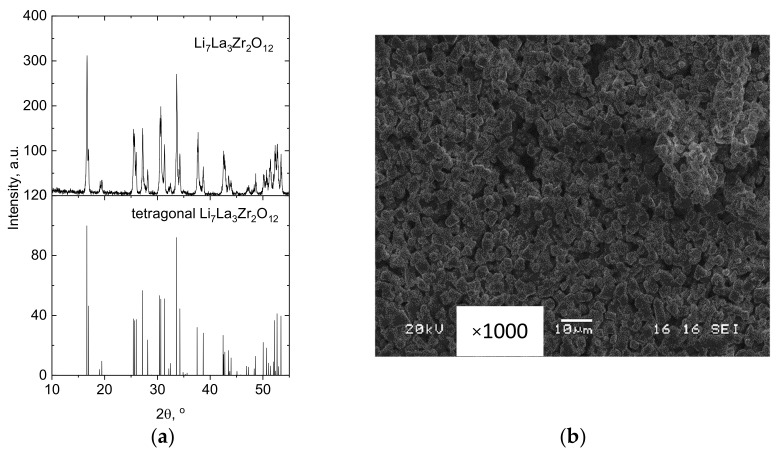
Characterization of LLZ powder: (**a**) XRD data; (**b**) SEM image.

According to XRD data, the obtained powder of LLZ was single-phase and had a tetragonal structure I41/acd [9], Figure 1a. The particles of LLZ powder have an irregular form with a size of ~3–5 µm (Figure 1b). The data obtained are in good agreement with the particle-size distribution of powder, which was previously determined by a laser-diffraction particle size analyzer [29].

The specific surface area of powder particles (S_BET_), according to BET analysis, was equal to 1.9 m^2^/g. The particle size (*d_av_*) was recalculated from BET data using formula (1) and amounted to 0.62 μm. Therefore, we can conclude that the ceramic grains presented in the SEM image (Figure 1b) are agglomerates of LLZ particles.

(1) $$d_{av}=\frac{6}{\rho \cdot S_{BET}},$$

where *ρ*—density of Li_7_La_3_Zr_2_O_12_, 5.106 g/cm^3^ [9].

As noted above (Materials and Methods), the isopropanol/acetylacetone (70/30 vol.%) mix was used as a dispersion medium for the suspension preparation based on LLZ powder. The measurement results of zeta potential and pH in LLZ suspension after ultrasonication at different times are presented in Table 1.

The initial value of the zeta potential was positive and equal to +6 mV at pH = 6.4 (Table 1). UST for 25 min leads to a significant change in zeta potential, while its value does not change (−6 mV). The further increase in UST time has no influence on the values of zeta potential. The change in zeta potential during ultrasonic treatment is insignificant and can be associated with some improvement in the solvation of particles by the dispersion medium. It can be assumed that the particle surface is activated during UST of suspension, which leads to the change in adsorption equilibrium. While the increase in UST duration leads to a change in equilibrium conditions and slight fluctuations in the suspension ionic composition, which are fixed by the change in pH (Table 1).

**Figure 2:**
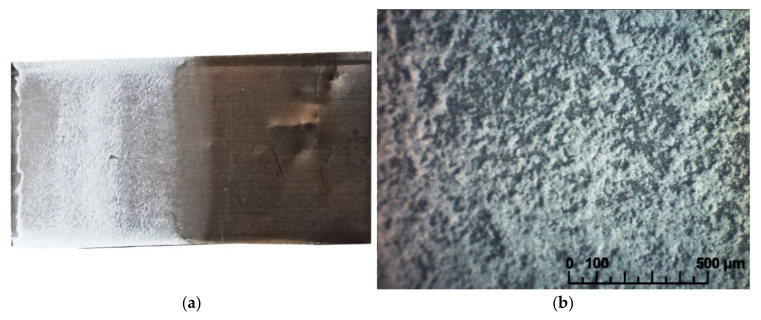
Surface of the dried LLZ coating obtained from a non-aqueous suspension of LLZ powder (10 g/L) during EPD on Ni-foil: (**a**) photo; (**b**) optical image.

A preliminary EPD experiment with the LLZ suspension obtained was carried out on the Ni-foil in the constant voltage mode of 80 V with a deposition time of 1 min. It was found that the continuous homogeneous coating was not formed (Figure 2).

Molecular iodine (0.4 g/L) was added to the LLZ suspension to carry out the stable EPD process and obtain the continuous coating. In some works [25,31,32], the introduction of iodine additives into suspensions of electrolyte powders is discussed. The value of zeta potential after the introduction of iodine into LLZ suspension did not change (−6 mV). However, the pH value shifted to the more acidic side from 6.4 to 5.4, which indicates a concentration increase in free protons in suspension due to the reaction of iodine with isopropanol. Proton generation in the reaction of iodine with alcohols was described in [33,34]. The continuous LLZ coating was obtained on the Ni-foil at a deposition voltage of 80 V and a time of 1 min from suspension with iodine addition. It should be noted that the current values during EPD from LLZ suspension with and without iodine additive differ significantly and are equal to 7.2 and 0.3 mA, respectively (Figure S1). Molecular iodine acts as a charging agent and leads to conductivity growth in the suspension.

### 3.2. Electrophoretic Deposition from LLZ Suspension on Ti Substrate

**Figure 3:**
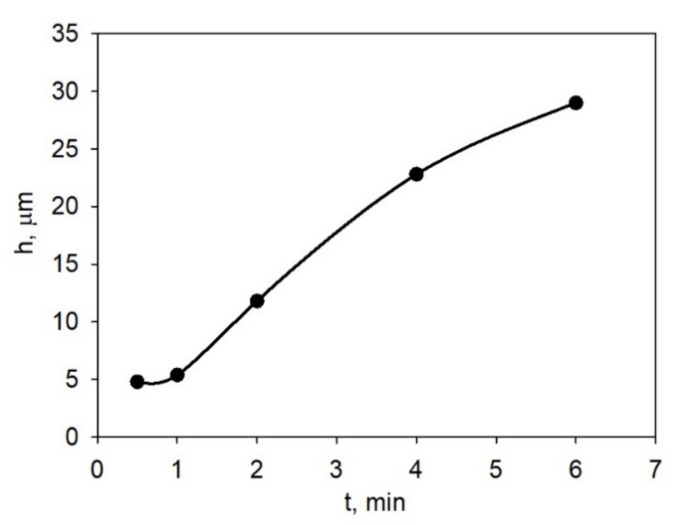
The thickness dependence of the dried LLZ coating on the time of EPD at a constant voltage of 80 V from LLZ suspension (10 g/L) with iodine addition (0.4 g/L).

To establish the EPD regime for obtaining continuous LLZ coatings without cracks and with the required thickness (~30 μm), the effect of deposition time on the film thickness at the constant voltage (80 V) from LLZ suspension with a concentration of 10 g/L and an iodine addition of 0.4 g/L was investigated. The dependence of the dried coating LLZ thickness on the EPD time at constant voltage is shown in Figure 3.

**Figure 4:**
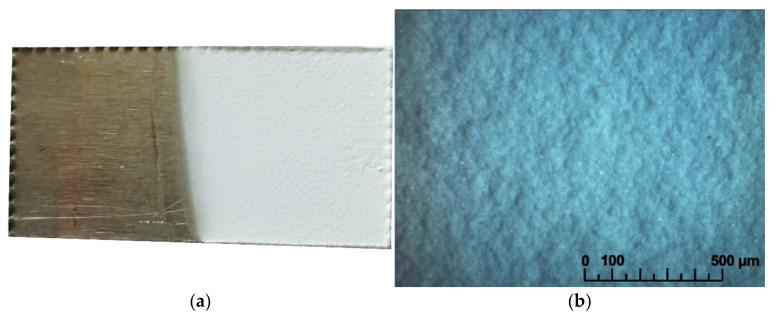
The surface of the dried LLZ coating obtained from non-aqueous suspension of LLZ powder (10 g/L) with iodine addition (0.4 g/L) during EPD on Ti-foil: (**a**) photo; (**b**) optical image.

An increase in the deposition rate in the initial period (up to 1.5 min) can be observed, then the deposition rate slowed down with the increase in film thickness and deposition time up to 6 min. To obtain continuous coatings (Figure 4) with a thickness of ~30 µm, the following deposition mode was chosen: voltage—80 V, time—6 min. As can be seen from Figure 4, a continuous, homogeneous LLZ coating without cracks was formed on the Ti substrate under the chosen conditions.

### 3.3. XRD and Microstructural Studies of LLZ Coatings

**Figure 5:**
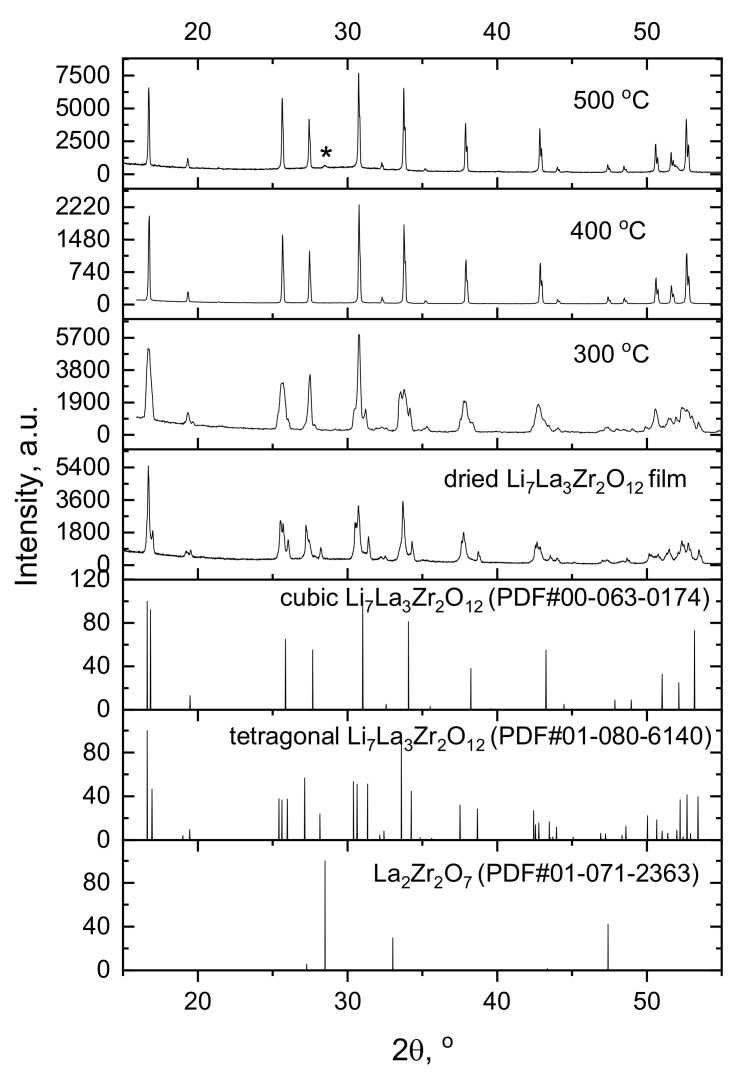
XRD patterns of LLZ films annealed at different temperatures. *—La_2_Zr_2_O_7_.

XRD patterns of the films annealed at various temperatures from 300 to 500 °C are shown in Figure 5. The dried film obtained by EPD has the same tetragonal structure (PDF #01-080-6140) as the initial LLZ powder. The XRD pattern of the film annealed at 300 °C shows the coalescence of some peaks, indicating the presence of a mixture of tetragonal and cubic modifications. The subsequent increase in the heat treatment temperature leads to the formation of the single-phase LLZ with a cubic structure (PDF #00-063-0174). The small impurity peak related to lanthanum zirconate (PDF #01-071-2363) is presented in the diffraction patterns of the LLZ film annealed at 500 °C. The formation of this impurity phase is commonly observed on solid electrolytes based on LLZ due to the partial volatilization of lithium oxide from samples [9].

According to the literature data [9,35,36,37,38,39], Li_7_La_3_Zr_2_O_12_ has a low-temperature cubic modification. In work [37], the low-temperature cubic modification was obtained by annealing tetragonal LLZ at 450 °C for 20 h in air. The phase transition from tetragonal to low-temperature cubic phase was reversible in air and was presumably due to the phase transforms because of the absorption of CO_2_ at 400–500 °C, and vice versa, the cubic phase transforms into the tetragonal one upon desorption of CO_2_. The low-temperature cubic modification of LLZ had a bulk conductivity of 1.3·10^−6^ S cm^−1^ at 25 °C. Larraz G et al. [38] suggested that the cubic phase formation at low temperatures can be related to the effect of water molecules incorporation and protonation via the H^+^/Li^+^ exchange mechanism on the garnet structure. In another study [39], low-temperature cubic modification of LLZ was obtained by the sol–gel method followed by annealing at 700 °C (20 h). It was found that this modification is unstable above 800 °C and has an ionic conductivity of ~10^−6^ S cm^−1^.

**Figure 6:**
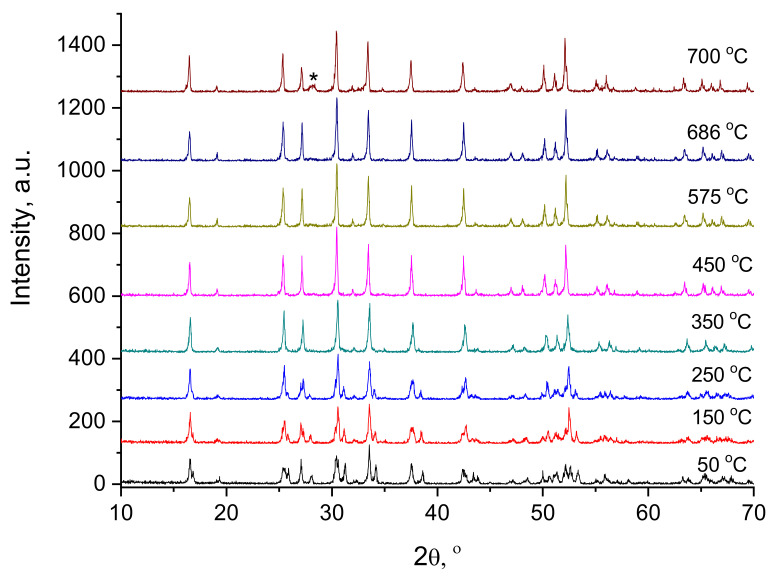
High-temperature XRD analysis of the tetragonal LLZ powder. *—La_2_Zr_2_O_7_.

A high-temperature XRD analysis of the obtained LLZ powder with a tetragonal structure was carried out (Figure 6). According to the obtained data, the phase transition from tetragonal modification to cubic modification occurs in the temperature range of 250–350 °C, and the well-formed cubic LLZ phase is observed at 450 °C. The smooth transition from tetragonal to cubic phase at temperatures above 350 °C was also observed in [36] during NMR studies of tetragonal LLZ. This structure change had no effect on the Li^+^ mobility, which retains a strictly Arrhenius character in the wide temperature range. According to high-temperature XRD data, the additional impurity peak of La_2_Zr_2_O_7_ is observed at 700 °C, which indicates the partial evaporation of lithium oxide from the studied powder [9]. While the appearance of this impurity in the films obtained is observed at lower temperatures (500 °C) with isothermal exposure (Figure 5), which can be related to the developed film surface and sample thickness.

**Figure 7:**
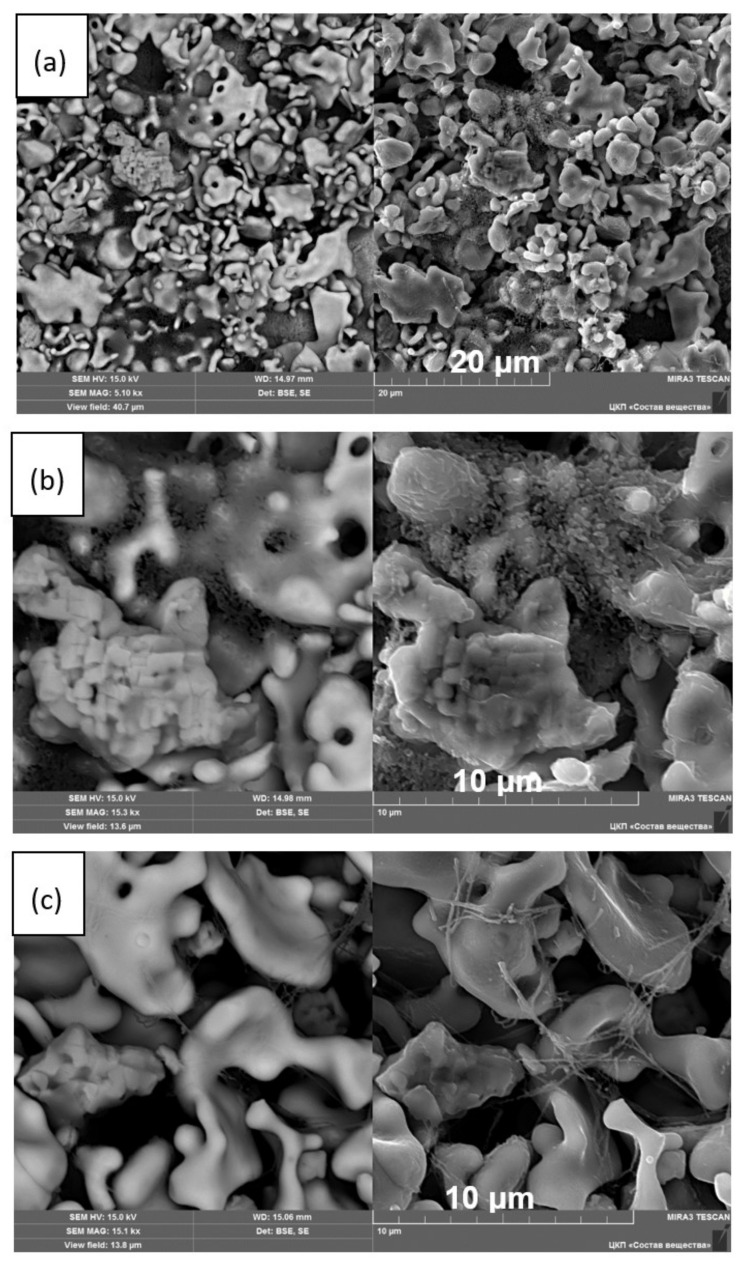

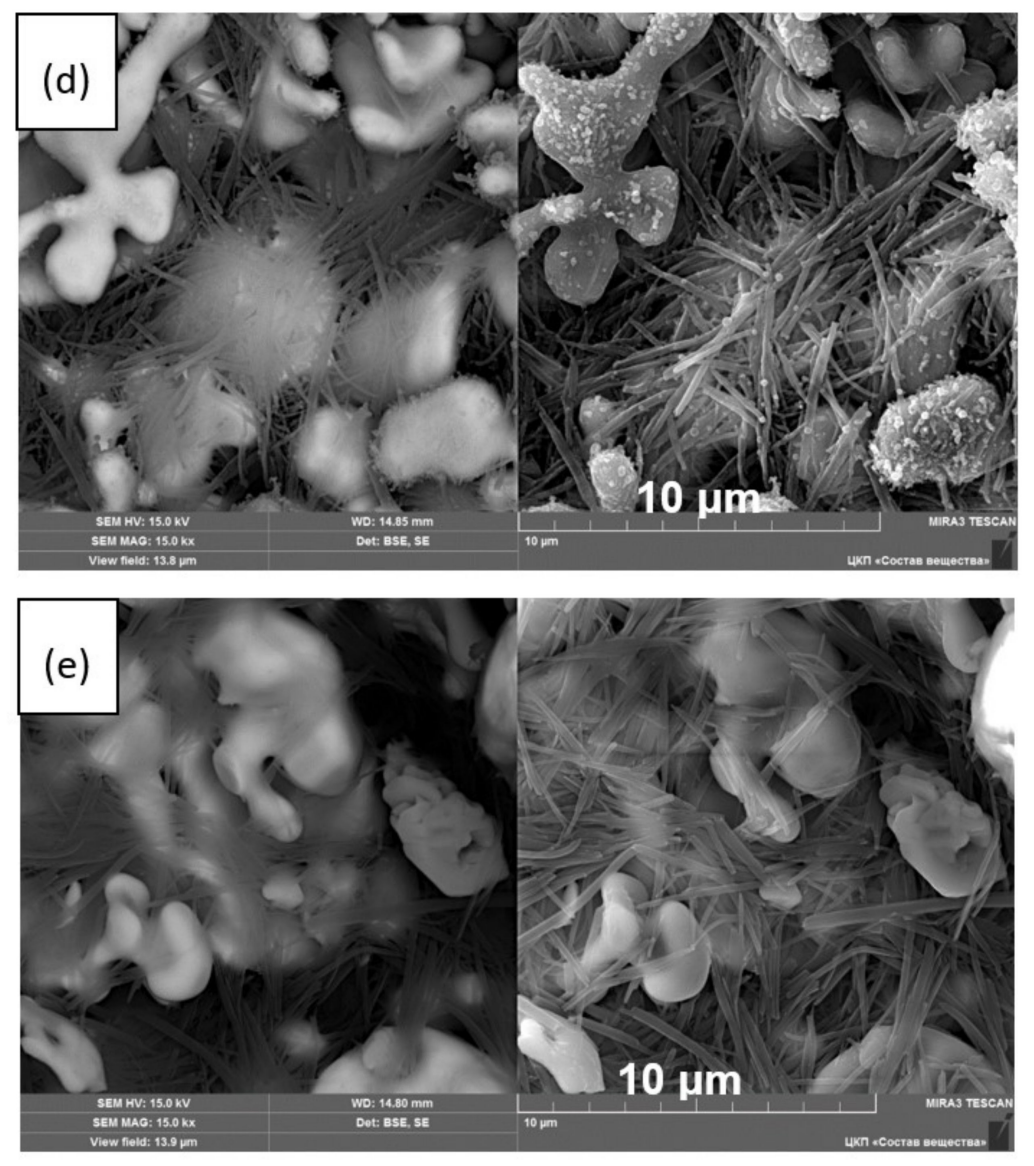
SEM images in BSE (left image) and SE (right image) modes of the LLZ films surface: dried (**a**,**b**) and annealed at 300 (**c**), 400 (**d**), and 500 °C (**e**).

**Figure 8:**
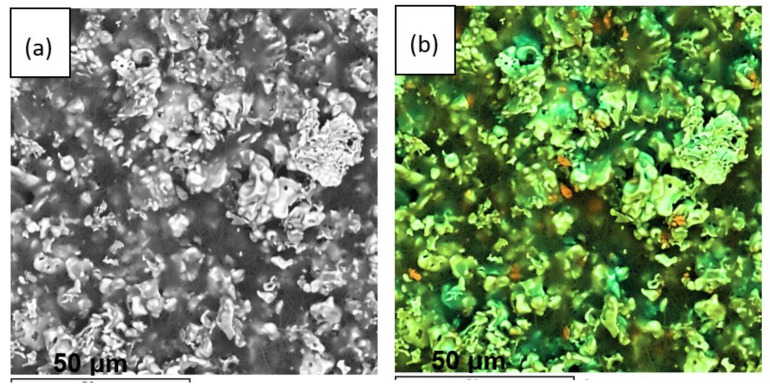

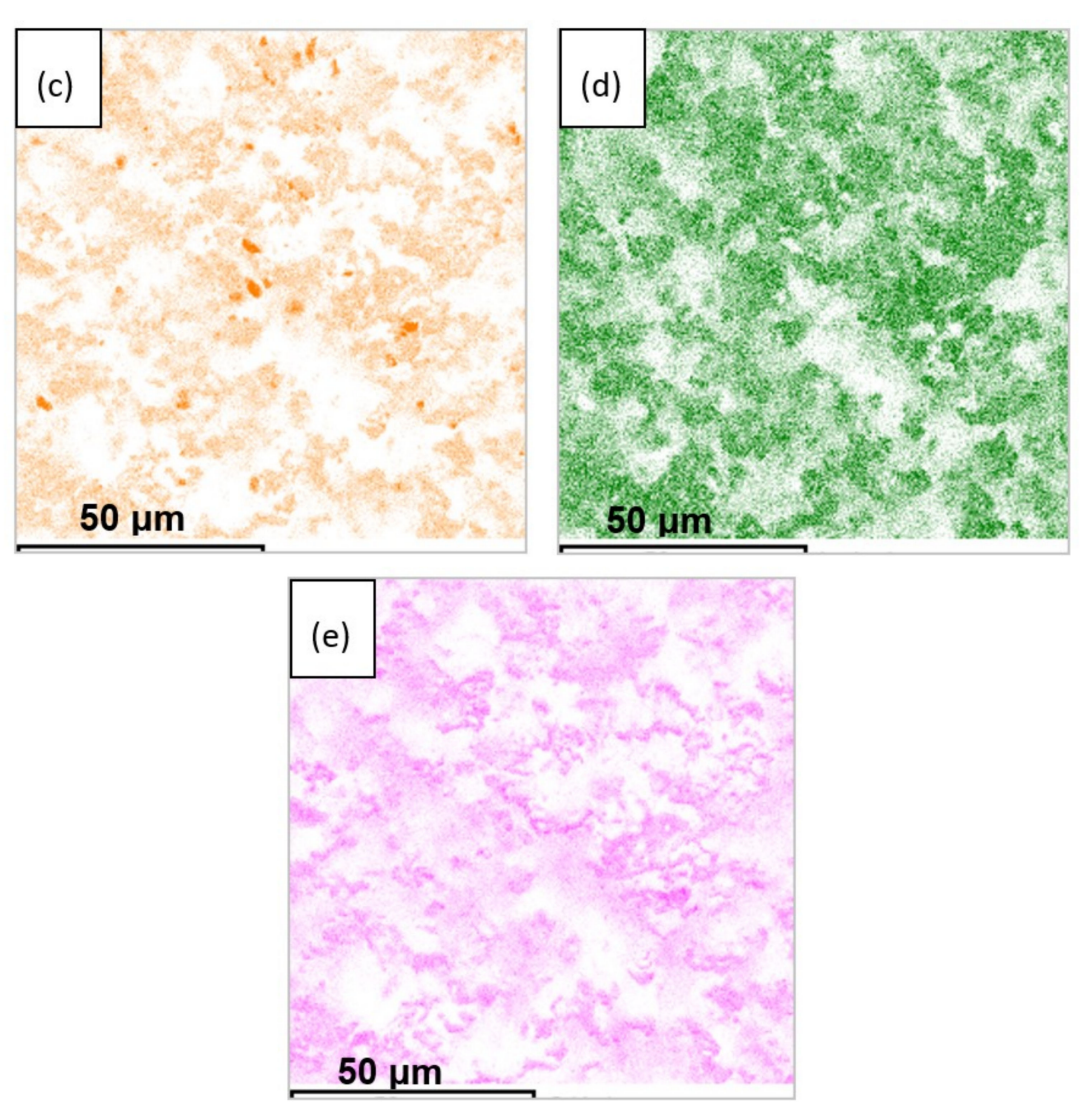
SEM micrograph of the LLZ film annealed at 500 °C (**a**,**b**) and element distribution maps for zirconium (**c**), lanthanum (**d**), and oxygen (**e**).

SEM micrographs of the LLZ films annealed at different temperatures are shown in Figure 7. The dried LLZ coating is characterized by a sufficiently developed surface; ceramic grains have an irregular shape and different grain sizes up to 10 μm. In some areas, the coating obtained by EPD contains agglomerates of submicron particles on the surface of the ceramic grains. Annealing at 300 °C leads to the disappearance of these particles and the formation of thin fibers (Figure 7c,d). The network of fibers increases and becomes denser after the annealing temperature growth of the films. To establish the phase composition of this fiber phase, elemental analysis of the film annealed at 500 °C was carried out (Figure 8). No additional elements indicating the interaction of the electrolyte film with the Ti substrate or air components were found. Only a small amount of impurity grains is present, which can be attributed to La_2_Zr_2_O_7_ according to the XRD data (Figure 5).

**Figure 9:**
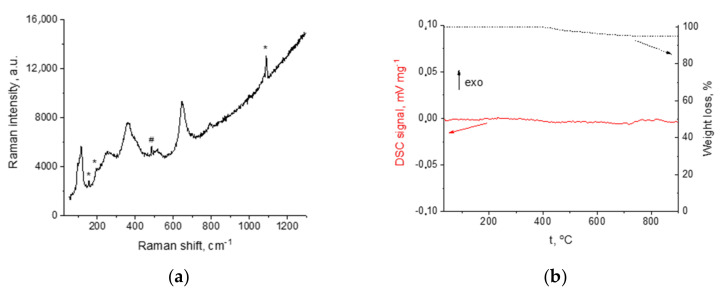
Raman spectra (**a**) and DSC curve (**b**) for LLZ film annealed at 500 °C. *—Li_2_CO_3_; #—La_2_Zr_2_O_7_.

Raman and DSC analyses of the film annealed at 500 °C were performed to determine the composition of the phase formed upon thermal treatment in air. According to Raman spectroscopy data (Figure 9a), the LLZ film annealed at 500 °C has a cubic structure [40] with impurity phases of lithium carbonate and lanthanum zirconate, which was also observed on XRD patterns. The DSC curves of the film obtained by electrophoretic deposition after its annealing at 500 °C do not show any thermal effects up to 900 °C, Figure 9b. A weight loss (4.8%) was observed for the sample studied. According to the work [38], such weight loss can be explained by H_2_O and CO_2_ evaporation. This phase formation was observed due to the contact of LLZ films with air atmosphere and during their annealing in air. It should be noted that the developed surface and small thickness of the obtained coatings lead to their higher reactivity with air components compared to bulk ceramic analogs.

### 3.4. Lithium-Ion Conductivity Studies of LLZ Coatings

**Figure 10:**
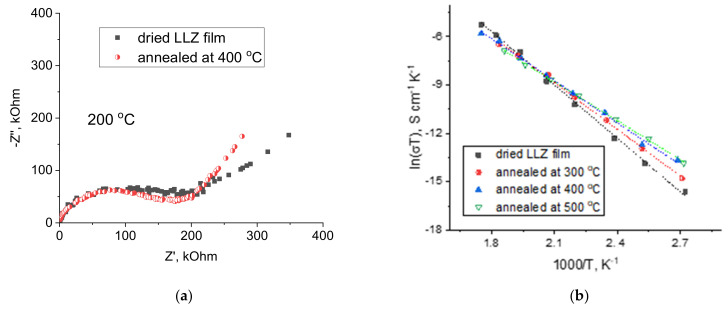
Impedance plots at 200 °C (**a**) and Arrhenius plots (**b**) for the total conductivity of dried LLZ film and films annealed at different temperatures.

Impedance plots of ceramic membranes after their heat treatment at different temperatures are similar and represent only one semicircle, which starts from zero at temperatures higher than 100 °C (Figure 10a). This semicircle is responsible for the total resistance of the solid electrolyte. A similar type of impedance plot was also observed for other films based on LLZ obtained by various methods [13]. It can be seen from the Arrhenius plots for the total conductivity of the studied samples (Figure 10b) that the film annealing up to 400 °C leads to insignificant conductivity growth of ceramic at 100 °C from 4.4·10^−10^ to 2.9·10^−9^ S cm^−1^ for dried LLZ film and annealed at 400 °C, respectively. At higher temperatures, all studied samples have similar values of total conductivity (~10^−6^ S cm^−1^ at 240 °C) due to the difference in activation energy. The activation energy of the conductivity of the dried sample is equal to 91.8 ± 2.3 kJ/mol, while the films annealed at 400 and 500 °C have lower values—72.3 ± 1.7 and 66.9 ± 0.7 kJ/mol, respectively. Therefore, it can be assumed that the formed fiber phase leads to a slight improvement in lithium-ion conductivity.

**Figure 11:**
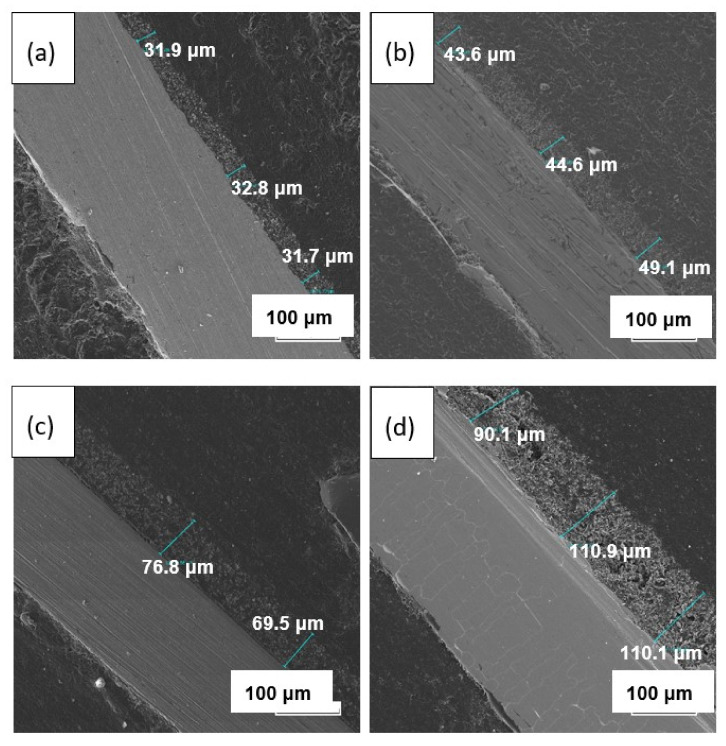
SEM images of the cross-section of the dried LLZ film (**a**) and films annealed at 300 (**b**), 400 (**c**), and 500 °C (**d**).

The cross-section of the LLZ solid electrolyte annealed at different temperatures is shown in Figure 11. With the annealing temperature increase, the gradual thickness growth of the dried film from 32 to 104 µm is observed. This behavior is associated with the formation of the additional fiber phase, which was observed on the film surface by SEM (Figure 7). Moreover, the film growth leads to the formation of a more porous film structure. While the thickness of the dried film after its annealing in an Ar atmosphere at 500 °C is equal to ~43 µm (Figure S2). This value is closer to the dried film and much lower compared to the thickness of the film annealed in an air atmosphere at 500 °C (104 µm). Based on the presented investigations, it can be concluded that the annealing of LLZ films obtained by electrophoretic deposition in an air atmosphere leads to their chemical reaction with air components.

## 4. Conclusions

The electrophoretic deposition method was applied to obtain the films of tetragonal Li_7_La_3_Zr_2_O_12_. The initial powder was characterized by BET, XRD, and SEM analyses. The composition of the LLZ suspension and EPD regime was developed to carry out the stable process of deposition and to obtain a continuous coating of LLZ on the Ni and Ti substrates. It was established that heat treatment of the deposited films leads to the phase transition of LLZ solid electrolytes from tetragonal to low-temperature cubic modification. According to the high-temperature XRD analysis, the process of this phase transition starts at 250 °C. According to XRD and SEM data, the films annealed at 500 °C have an impurity phase—La_2_Zr_2_O_7_. Moreover, the formation of additional phases in the form of fibers was observed in the SEM images of the LLZ film annealed at 300 °C. The temperature growth leads to an increase in fiber content and film thickness from 32 to 104 μm for the membranes dried and annealed at 500 °C, respectively. This phase was not detected by XRD or element distribution analysis. Therefore, Raman spectroscopy and DSC methods were used to establish the phase composition of the fibers. The DSC curve of the solid electrolyte membrane annealed at 500 °C does not have any exo- or endo-effects, but according to TG, the weight mass of the sample was observed, which can be associated with H_2_O and CO_2_ evaporation. The film studied has the structure of cubic LLZ with the small addition of La_2_Zr_2_O_7_ and Li_2_CO_3_, according to Raman spectroscopy data. The conductivity of the obtained lithium-ion membranes was determined by the impedance spectroscope method. The total conductivity of the deposited LLZ film is equal to 4.4·10^−10^ S cm^−1^ and 1.8·10^−7^ S cm^−1^ at 100 and 200 °C, respectively. The growth of the annealing temperature up to 500 °C leads to the insignificant conductivity increasing (2.9·10^−9^ S cm^−1^ and 3.6·10^−7^ S cm^−1^ at 100 and 200 °C, respectively). Thus, it can be concluded that films of solid electrolytes based on LLZ can be obtained by electrophoretic deposition. However, their heat treatment in air leads to the formation of additional fiber phases and insignificant conductivity growth.

## Figures and Tables

**Table 1:** Influence of ultrasonic treatment time on zeta potential and pH value in LLZ suspension.

UST, min	Zeta Potential, mV	pH
5	+6	6.4
25	−6	7.1
125	−6	6.4

## Data Availability

Not applicable.

## Supplementary Materials

The following supporting information can be downloaded at: https://www.mdpi.com/article/10.3390/membranes13050468/s1, Figure S1: The time dependences of current during EPD in Li_7_La_3_Zr_2_O_12_ suspension without and with iodine addition (at a constant voltage on the electrodes of 80 V); Figure S2: SEM image of the cross-section of LLZ film annealed at 500 °C in Ar atmosphere.
