# Supplementary material for: Electrophoretic Deposition and Characterization of Thin-Film Membranes Li7La3Zr2O12
Source: Membranes (Basel). 2023 Apr 27;13(5):468. doi: 10.3390/membranes13050468 (PMC10223024; doi:10.3390/membranes13050468)
Supplement: Supplementary file 1 [file membranes-13-00468-s001.zip › membranes-2331142-supplementary.pdf]

Supplementary materials

Electrophoretic Deposition and Characterization of Thin-film Membranes $Li_7La_3Zr_2O_{12}$

Efim Lyalin [1], Evgeniya Il'ina [1], Elena Kalinina [2,3,*], Boris Antonov [1], Alexander Pankratov [1] and Danil Pereverzev [4]

[1] Laboratory of Electrochemical Power Sources, Institute of High Temperature Electrochemistry, Ural Branch of the Russian Academy of Sciences, 620990 Yekaterinburg, Russia; efim.lyalin.2013@inbox.ru (E.L.); ilyina@ihte.uran.ru (E.I.); antonovbd@ihte.uran.ru (B.A.); a.pankratov@ihte.uran.ru (A.P.)

[2] Laboratory of Complex Electrophysic Investigations, Institute of Electrophysics, Ural Branch of the Russian Academy of Sciences, 620016 Yekaterinburg, Russia

[3] Department of Physical and Inorganic Chemistry, Institute of Natural Sciences and Mathematics, Ural Federal University, 620002 Yekaterinburg, Russia

[4] Laboratory of Solid State Ionics, Institute of Solid State Chemistry, Ural Branch of the Russian Academy of Sciences, 620108 Yekaterinburg, Russia; danil_per@mail.ru

* Correspondence: jelen456@yandex.ru; Tel.: +7-343-267-87-82

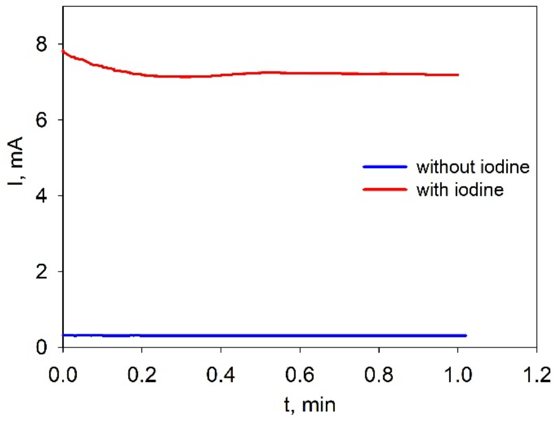

Figure S1. The time dependences of current during EPD in $Li_7La_3Zr_2O_{12}$ suspension without and with iodine addition (at a constant voltage on the electrodes of 80 V).

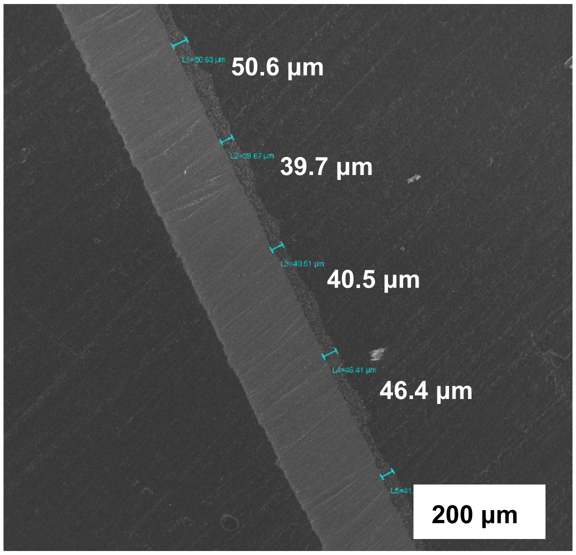

Figure S2. SEM image of the cross-section of LLZ film annealed at 500 °C in Ar atmosphere.
